# Facilitators and Barriers in Integrated Social Care for Families Facing Multiple and Complex Problems: A Scoping Review

**DOI:** 10.5334/ijic.7768

**Published:** 2024-08-07

**Authors:** Marcel van Eck, Roelof Ettema, Mariëlle Cloin, Tine Van Regenmortel

**Affiliations:** 1 Tilburg School of Social & Behavioral Sciences, Tilburg University, Tilburg, The Netherlands; 2 Research Group Personalized Integrated Care, Institute for Nursing Studies, Utrecht University of Applied Sciences, the Netherlands; 3 Faculty of Social Sciences –HIVA, University of Leuven, Leuven, Belgium

**Keywords:** families with multiple and complex problems, multi-problem families, integrated social care, integrated care, multi-agency working, interdisciplinary collaboration, fragmentation of care

## Abstract

**Introduction:**

Families with multiple and complex problems often deal with multiple professionals and organizations for support. Integrated social care supposedly prevents the fragmentation of care that often occurs.We identified facilitators and barriers experienced by families receiving integrated social care and by the professionals who provide it.

**Method:**

We performed a scoping review following Arksey and O’Malley’s framework, using the following databases: PsycINFO, Web of Science Core Collection, Psychology and Behavioral Sciences Collection, CINAHL, PubMed, and Medline. Furthermore, conducted a thematic analysis. The results were divided into facilitators and barriers of integrated social care.

**Results:**

We identified 278 studies and finally included sixteen in our scoping review. We identified facilitators, including: linking formal care with informal networks, promoting collaboration among professionals e.g., working in pairs, and professionals autonomy. We identified barriers, including: time constraints, tasks outside professionals’ expertise, along with resistance to integrated collaboration among organizations. These findings can enhance the advancement of social integrated care as a promising approach to support families facing multiple and complex problems.

**Conclusion:**

To empower families, integrated social care requires a systematic approach based on trust. It involves coordinated care, shared decision-making, informal networks and the participation of all family members, including children.

## Introduction

Families facing multiple and complex problems often deal with severe, chronic difficulties and multiple stressors. Problems frequently accumulate and interact with each other in different areas of life are, for example, poor family functioning, parenting problems, (mental) health, financial problems, and substance abuse [1]. In the literature, various terms are used to identify families that encounter such difficulties, for example, multi-problem families; multi-stressed families; multi-crisis families; and multi-assisted families [2]. Although the terms slightly differ in nature, it is generally agreed that they concern families experiencing multiple and complex problems [3,4]. Although all these names serve the same target group. In this study we used the following definition for families with multiple and complex problems: “families who suffers long-term combined socio-economic and psychosocial problems, which manifest themselves in various areas within the family” [5].

Because of the complexity and interconnectedness of the problems these families encounter, support from a single professional is often insufficient [3]. As a consequence, families often receive support from social workers, youth professionals, counsellors, (mental) healthcare providers, psychologists, psychiatrists, teachers, and nurses from different service organizations. These professionals often tend to work from their own area of expertise and therefore focus on a specific problem [6]. While it would be preferable for social care to be inherently integrated, this often is not the case highlighting the fragmented nature of social care services. Social care often operate in silos with limited coordination and collaboration among different service providers and sectors [6].

This is considered a risk since it is well known that care is not always well coordinated and can lead to fragmentation of care and support in this case for families already much in need of support. This fragmentation means that professionals do not know each other what they are doing and that they work past each other [7,8,9]. As a consequence fragmentation jeopardizes successful treatment, decreases client satisfaction, and limits the effectiveness of the support [8]. Furthermore, with multiple professionals each focusing on a specific problem, there is a risk that some issues may be overlooked [10]. This has negative consequences for the quality of care, disproportionate use of scarce services, and high costs for social care systems [8,11,12,13]. Integrated care in general is suggested as a promising approach to counteract the fragmentation of care [14], despite the use of different operationalizations and definitions [15,16,17,18].

A frequently used version of integrated care is as follows: “An approach to strengthen people-centered health systems through the promotion of the comprehensive delivery of quality services across the life-course, designed according to the multi-dimensional needs of the population and the individual and delivered by a coordinated multidisciplinary team of providers working across settings and levels of care” [13]. In this study we applied this in social care: Integrated Social Care. Here, the focus is on integrated care among social services, distinct from for instance its integration with medical care, primarily to enhance the delivery of the complex social care for families facing multiple and complex problems.

Much is known in the literature from barriers and facilitators about integrated care in general, with a focus on medical care [19]. Studies have for instance shown that integrated care is associated with improved quality of care and increased client satisfaction [10]. We could not identify such studies for integrated social care. Therefore, we focussed on barriers and facilitators of integrated social care departing from the above-mentioned definition of integrated care. Integrated social care resonates with the definition of integrated healthcare, sharing core elements and similarities such as comprehensiveness, (care) coordination, cooperation between professionals and organizations, partnership, and holism, to improve care, health and well-being [13]. Furthermore, integrated social care in its broadest sense can come from a variety of professional sources, as well as informal resources such as family and friends [8].

Although there is research into one or more factors that contribute to integrated social care, knowledge about this is also fragmented. For instance, research focuses specifically on youth care rather than integrated care with other domains within social care [1]. Other research focuses specifically on care coordination for vulnerable families which is only an aspect of integrated social care. [20]. Comprehensive knowledge about barriers an facilitators for social integrated care for families with multiple and complex problems so far is lacking. In addition, existing literature pays attention to integrated care, but not specifically to the social domain of integrated care. Therefore, study aims to identify facilitators and barriers for families receiving integrated social care and for professionals, policymakers and researchers to improve providing integrated social care in practice and research.

We formulated the following research questions: What are facilitators and barriers for integrated care within the context of social care for families facing multiple and complex problems who receive this care and for professionals who provide it?

## Methods

We performed a scoping literature review following the PRISMA guidelines for scoping reviews [21]. Our aim was to provide an overview of the existing literature in order to identify these facilitators and barriers [22].

We conducted a literature search, selection, and synthesis of existing knowledge to chart key concepts, types of evidence, and research gaps related to facilitators and barriers in integrated social care, as perceived by both families and professionals [22]. For this we used the six-stage process of the scoping review framework originally developed by Arksey and O’Malley [23], with additional recommendations from Levac et al. [24].

### Stage 1. Identifying the research questions fewer

To answer the research question, we identified facilitators and barriers in integrated social care for families and professionals and we provide an overview of the relevant literature and considered the purpose of this scoping review, as described in the introduction.

### Stage 2. Identifying relevant studies

To identify the relevant literature, we searched the following databases: PsycINFO, Web of Science Core Collection, Psychology and Behavioural Sciences Collection, CINAHL, PubMed, and Medline in the period December 2022-October 2023.

The search was performed with an alternate combination of Boolean search operators (AND/OR; Table 1). We supplemented this search with manual searches of reference lists of the identified articles. In our scoping review, we employed the PICO framework (Population, Intervention, Comparison, Outcome) [25] to construct the search string. Since this study does not involve a comparison between interventions, the ‘C’ component was excluded. Additionally, trial and error revealed that including the ‘O’ (Outcome) component resulted in less relevant articles, leading to its exclusion as well. The search rule was composed by the first author MvE and the second author RE.

**Table 1:** Selection criteria.


INCLUSION CRITERIA	EXCLUSION CRITERIA

– Published during the period 1995–2023	– Focus exclusively on adults or older people rather than families or healthcare solely

– Studies focussed specifically on integrated care, as defined in the introduction, within the context of social care	– Studies outside North America, Europe and Australia

– Studies focused on families with multiple and complex problems, as defined in the introduction	– Editorials and opinion pieces, commentaries, systematic reviews

– Studies including different forms of interventions within integrated social care	– Informal sources, grey literature

– Studies providing outcomes that highlight both facilitators and barriers to integrated care for both families and professionals	

– All different kind of studies	


See appendix 1. Database search overview.

### Stage 3. Study selection

The data screening program Rayyan QCRI was used to select the articles [26]. The selected articles were arranged by author(s), the year of publication, and the contribution to the research question. The results were mapped at the family and professional level to elucidate what is currently known about integrated social care for families experiencing multiple and complex problems. For both levels, we used inductive reasoning to identify overarching themes. The definition of integrated social care as defined in the introduction serves as a central concept, and so does the concept of families with multiple and complex problems. The inclusion and exclusion criteria in Table 1 have been drawn up to select the articles.

The studies were selected and categorized by MvE. Any doubts about whether an article should be included were discussed with RE.

### Stage 4. Charting the data

We first mapped the first author, the year of publication, the study location, and the study design. The results were then classified into facilitators and barriers. Table 2 provides a summary of the data:

**Table 2:** Summary of the data.


	STUDY DESIGN	COUNTRY	FACILITATORS FOR FAMILIES	BARRIERS FOR FAMILIES	FACILITATORS FOR PROFESSIONALS	BARRIERS FOR PROFESSIONALS

**Serbati, et al, 2016** [29]	Pre- and post-test design (qualitative and quantitative)	Italy	– the importance of multidimensional assessments and interventions	gap between social services – parents often feel blamed and excluded from decision-making – parents are confused by a system that seems to hold power over them	– multi-professional decision-making	

**Eastwood et al, 2020** [30]	Realist evaluation	Australia	– involvement of the whole family – families participating in decision-making – cultural sensitivity – flexible professionals – shared decision making		– co-location – motivation of professionals and services increase collaboration – no strict referral criteria	– collaboration leads to difficulties in information sharing

**Eastwood et al, 2020** [31]	Realist evaluation	Australia	– adaptability to intensity of families’ fluctuating support – trust between family and professional leads to a successful working relationship – shared decision-making between professional and family members – favourable inter-personal relations between clients and professionals – culturally-appropriate, trauma-informed care – flexibility of accessibility and service navigation	– distrust of welfare services by family members	– favourable inter-personal relations between service providers – absence of strict referral criteria – creation of trusting relationships between service-providers	– mutual competition between organizations – underdeveloped pathways for intra- and interagency collaboration – fragmented service environment – professional autonomy can lead to a high degree of responsibility, which can create a risk of burnout – difficulty maintaining healthy boundaries empathy and professionalism amongst professionals – persistent silos in healthcare and systemic resistance to collaboration – professional autonomy

**Tennant et al, 2020** [32]	Realist evaluation	Australia	– building trust between professionals/family members – likeable and approachable: ’a safe person’ – meeting clients on their own terms – quickly demonstrating staff effectiveness – client empowerment		– shared learning amongst collaborating professionals – leveraging other family members – social and organizational relationships – mutual respect amongst professionals – co-location of professionals – multidisciplinary and/or interagency staff – flexible service by professionals – knowledge transfer between staff working together – advocacy for other professionals or agencies	– difficulties relating to privacy – care-coordinators combining their interactions with child welfare workers can result in conflicts with families – flexibility leads to burnout symptoms amongst professionals – professionals who depend on other services can jeopardize the relationship with families

**Nooteboom et al, 2020** [33]	Qualitative	Netherlands	– holistic, family-centred approach – shared decision-making – jointly prioritize needs and focus of support – an up-to-date care plan – clarity, tasks, and responsibilities – co- located professionals – a care coordinator – frequent evaluation – familiarity between professionals through interprofessional collaboration – accessibility of professionals	– cultural and generational differences in talking about problems (by involving social networks) – overburdening social networks (by involving them) – not all parents feel the need to use theirs social networks – too many treatment goals lead to overburdening of parents – long waiting lists – lack of clarity of services – perceived limited freedom of choice; differences in appropriate support between professionals – parents feel uncomfortable about sharing personal information – warm handoffs – lack of availability of professionals		

**Morris 2013** [34]	Qualitative	United Kingdom	– involving family narratives in support of practical help – understanding the family results in greater engagement with services – understanding the everyday reality of families	– withholding of information by families – not recognizing the challenges faced by families	– working with family groups instead of individual family members	

**Bachler et al, 2016** [35]	Pre/post-naturalistic	Austria/Germany	– opportunity to develop psycho-social skills by establishing treatment expectation – developing working alliance (therapist and family) – systemic, family-wide approach	– less goal-directed collaboration – sufficient self-efficacy in solving problems by family itself	– insufficient time for contemplation amongst professionals – not being aware of family-related psychosocial problems	

**Onyskiw et al, 1999** [36]	Descriptive/evaluative	Canada	– informal support, accepting, non-threatening, non-judgemental, and help for coping with stressors – multidisciplinary teams appreciated by clients – families found education and support groups beneficial	- home visits not always seen as positive by clients - project operated during business hours		

**Sousa 2005** [37]	Qualitative/explorative	Portugal	– supporting role of the social network – informal network guide to other support – networking approach enabling dealing with crisis – informal network has more weight than formal network	– families less reciprocal in social contacts – over-involvement of professionals leads to the delegation of responsibility from families to professionals		

**Lawick et al, (2008)** [38]	Qualitative	The Netherlands	– humour – feedback between families and professionals – home visits – collaborative position between families		– working with ’multi-stressed’ families is overwhelming for therapists	

**Bachler et al, (2017)** [39]	Naturalistic	Austria	– qualitative collaboration between professionals and families leads to high treatment outcome expectancy and reduces stress – supporting family-wide approach	– family members do not maintain or improve collaboration – hopelessness in clients leads to reduced treatment outcomes – increased child development risks in families with low socio-economic status (SES)		

**Thoburn et al, (2013)** [40]	Ethnographic	United Kingdom	– whole holistic family approach – availability of a second key worker (one for the child and one for the parent[s])	– lack of flexibility in approach by professionals – ambivalent trust in the professional – crucial aims are not achieved	– access to specialist and statutory support services – flexibility of intensity and case duration – high level of supervision and consultation for professionals – multi-agency partnerships – range of different approaches	

**Nooteboom et al, (2020a)** [41]	Qualitative	The Netherlands	– broad assessment across different areas of life – early consultation and involvement of informal networks and schools		– professionals see home visits as advantageous – frequent evaluation support process and collaboration with families and professionals – a support plan focused on the future – importance of timely recognition of risks and needs – multidisciplinary expertise within teams – agreements about tasks, roles, and responsibilities at the organizational level – accessibility and availability for families – autonomy of professionals and tailored support – professionals work in pairs – familiarity with other professionals through co-location – warm handoff professionals – coordination of care – jointly discuss focus of support in multidisciplinary teams	– lack of knowledge of dealing with different problems amongst professionals – difficulties with family privacy re. sharing of information – difficult to determine when to scale support up or down – resistance of families to restrictive support in scaling up – too much involvement with family – case discussions too crisis-orientated – prioritizing problems – barriers to interprofessional collaboration – risk of too much support regarding the problem(s) – high work pressure for professionals – risk of professionals working outside their expertise – professionals dealing with unclear tasks, roles, and responsibilities – waiting lists for access to social care; professionals experience difficulties in assessing crisis situations

**Sousa & Rodrigues (2009)** [42]	Qualitative	Portugal	– partnership between family members and professionals	– fragmenting support formal and informal network – difficulties families in reciprocal relationships		

**Nadeau et al, (2012)** [43]	Qualitative Participatory	Canada			– regular exchanges to resolve tensions and promote collaboration between teams – formal mechanisms for communication – clear referral procedures to increase stability in teams – possibilities for informal communication between workers – opportunities for clinical discussion	– shifts of power and a loss of privileges that upset relationships between clinicians and managers – lack of knowledge of other institutions

**Tausenfreund et al, (2014)** [44]	Prospective one-group repeated measures outcome	The Netherlands	– dual key worker approach: one for the parents and one for the children	– prematurely stopping support – high stress levels in families

- Facilitators of and barriers to integrated social care amongst families with multiple and complex problems;
- Facilitators of and barriers to integrated social care amongst professionals.

### Stage 5. Collating, summarizing, and reporting results

A critical appraisal of qualitative and quantitative research was carried out using Treloar et al. Butcher et al. [27,28 Appendix 2]. We than performed a qualitative thematic analysis by coding the results according to different themes. We searched for recognizable patterns in the data and distilled seven themes with regard to families and seven themes with regard to professionals. See Table 2 for the facilitators and barriers within the two sets of seven themes.

### Stage 6. Consultation

The content of the studies was assessed and discussed with two authors (MC and TvR) who have expertise in this area.

## Results

**Figure 1:**
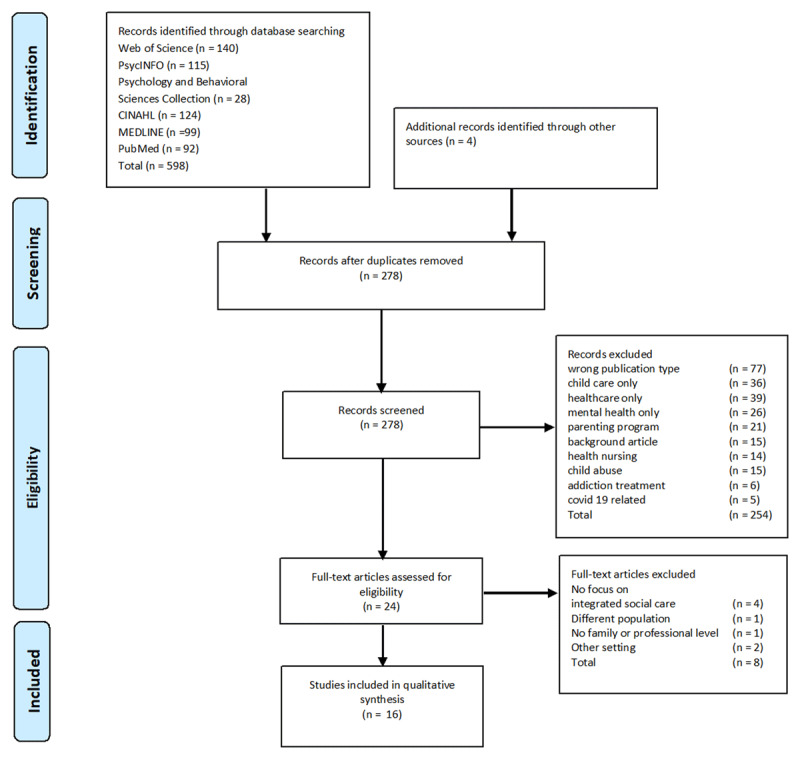
Flow chart of the study.

A total of 598 articles were initially identified, and after removing the duplicates, 278 remained. Four articles were found through a manual search. After screening titles and abstracts, 254 were excluded. The 24 remaining articles were read in full and eight articles were excluded because they did not fit the inclusion criteria. Finally sixteen articles were selected. (Figure 1).

The data from the articles comprised facilitators of and barriers to integrated social care amongst families and professionals (see Table 2).

### Facilitators of and barriers to integrated social care amongst families

The studies identify a large number of facilitators and barriers that influenced the reception of integrated social care amongst families experiencing multiple and complex problems. By ‘amongst families’, it is meant that these barriers and facilitators are described from the perspective of families. We identified the following seven principal themes.

#### (Mis)match between families and social services

The gap between the real-life experience of families and the world of social services acts as a barrier to integrated social care. This can leave parents feeling confused and powerless [29], leading to mistrust [30,31,32] and negatively impacting the acceptance of integrated social care. Parents also perceive a limited freedom of choice in the support they receive [33], leading to a diminished sense of control.

Understanding the daily lives of families is considered to be facilitative [34] because engaging with families on their terms and beyond professional duties helps bridge the gap between their lived experience and the world of social services [32]. However, professionals may struggle to balance their responsibilities and display empathy, which can be a barrier [31].

Understanding the daily lives of families experiencing multiple and complex problems and offering practical help provided is considered a facilitator [34] because the families feel that the professionals are meeting them on their own terms [32]. Also, the literature indicates that professionals who show their appreciation to family members by acting outside their duties strengthen the collaboration [32], though it can be difficult to delineate the dividing line between their professional role and the degree of empathy required [31].

#### A relationship built on trust and collaboration

A facilitator of integrated social care is a collaborative relationship between family members and professionals, as well as formal and informal partners. Relationship-building takes time, particularly at the beginning of the support process [35]. Family members are not always immediately open to integrated social care, which acts as a two-way barrier [35]. Facilitators strengthening a collaborative relationship from the perspective of the family are an informal, accepting, non-threatening, and non-judgemental attitude from the professional [36]. Several of the studies point out that a relationship built on trust between professionals and families is a crucial facilitator [31,32,34,35,37]. Families appreciate professionals who are likeable and approachable [33], who exhibit an informal, accepting, non-threatening, and non-judgemental attitude, who address problems promptly, and who help to reduce stress [36]. It should also be noted that, because building relationships takes time, family members may be resistant to the integrated social care approach, which can create a barrier for professionals [35].

Another specific facilitator for integrated social care and collaboration is asking for feedback; this helps families to take the initiative and make a commitment to change. Humour can put problems into perspective and strengthen collaboration [38].

#### Hope and self-reliance

Professional support that helps families to develop a positive outlook and a sense of self-reliance and control are facilitative because such attributes reinforce collaborative goal-setting and the likelihood of positive outcomes. However, feelings of hopelessness act as a barrier to successful integrated social care [35].

#### Addressing problems in multiple areas of life

Integrated social care aims to address problems across various areas of life simultaneously. The research suggests that when this happens, it facilitates positive experiences of integrated social care, both for families and professionals [29,33,35,39,40,41].

However, the process presents challenges for families and professionals in terms of the prioritization and management of problems [41]. Additionally, too many treatment goals can overburden parents and lead to a decline in motivation and self-efficacy [33,34].

#### Systemic approach within the family and the informal social network

An important facilitator of integrated social care is a systemic approach where the family constitutes a system rather than a collection of individuals [33,35,40]. Both parents and professionals consider a systemic approach to be an important ingredient of integrated social care [33,41]. Various studies reveal that a social network approach involving other stakeholders leads to successful integrated social care, but an informal network can also be facilitative [37]. Thus, informal social support (e.g., family and friends) can relieve stress and provide support by assisting in the development of personal coping and parenting skills [36].

For the family, an informal network might be a more important source of support than the formal one, though a combination of the two makes it that much easier to overcome adversity [37]. Several of the studies argue that family members can benefit from building a reciprocal relationship within an informal network by involving family members, friends, or neighbours alongside the formal setup [32,35,42]. Informal networks can offer emotional support, which then creates a space wherein families become more open to other forms of help [38].

A potential barrier to informal networks includes difficulties in maintaining reciprocal relationships with others [42] and overburdened social ties. Moreover, parents do not always perceive informal networks as beneficial [33].

In turn, the extensive involvement of professionals in the social network can be a barrier because it can place too much responsibility on them; consequently, ownership does not reside within the families themselves or their personal networks [37].

The literature demonstrates that a systemic approach, involving all family members rather than individuals, is a key facilitator of integrated social care [33,35,40]. This is recognized by both parents and professionals [33,41]. Many of the studies argue that informal networks are an essential aspect of integrated social care [30,33,34,35,37,39,40,41,42]. Emotional support from family members, friends, and neighbours helps to create openness to other forms of help [38], guide families towards formal support [38], provide stress relief, and enhance coping and parenting skills [36]. Establishing mutual relationships within the informal network is a key aspect of integrated social care [32,35,42].

However, families may have limited or unsupportive social networks [42]. Preventing the overload of social networks can also be a challenge, and not all parents perceive them as beneficial [33]. Extensive involvement of professionals in the social network can hinder familial ownership and a sense of responsibility [38]. Finally, the fragmentation of care amongst formal and informal stakeholders is recognized as a barrier to successful integrated social care [42].

#### Shared decision-making

Several of the studies reveal that shared decision-making is an important facilitator from the family perspective [30,31,33]. Prioritizing problems with the professionals and up-to-date care plans are facilitative for many parents [33,41]. The professionals can then discuss the needs of the families in a comprehensive manner [29].

#### Home visits

Home visits create opportunities for successful integrated social care, especially when the professionals involved take a collaborative and egalitarian position [38,41]. Notwithstanding, home visits can sometimes cause discomfort and lead to a loss of control on the part of the families [36].

### Facilitators of and barriers to integrated social care amongst professionals

We also identified a large number of both facilitators and barriers that influenced the reception of integrated social care amongst professionals. By ‘amongst professionals’, it is meant that these barriers and facilitators are described from the perspective of professionals. There are seven principal themes.

#### Interprofessional collaboration

Shared professional learning can serve as a facilitator [32], along with multi-professional decision-making [29,31]. Warm handoffs between professionals are perceived as a facilitator of integrated social care from the perspective of parents and professionals [33,41]. Meanwhile, case discussions that are overly focused on crises [41]; difficulties in interprofessional collaboration [41]; privacy issues regarding the sharing of information [30,32,33]; and a lack of familiarity with other institutions can form barriers to interprofessional collaboration [43]. Also, in many cases, parents have reported a lack of clarity in service provision and the specific demands of organizations [33]. Professionals who have to compromise with child welfare workers can come into conflict with parents, and this in turn can hinder collaboration [32].

Shared professional learning and multi-professional decision-making facilitate the provision of integrated social care [29,31,32,34], though case discussions that prioritize crises can be a barrier to both professionals and parents [41]. Difficulties in interprofessional collaboration, which may arise from professionals having to operate within separate systems and cultures, hinder the provision of integrated social care [41], as do privacy concerns regarding information-sharing amongst professionals [30,32,33] and a lack of familiarity with other services or organizations [43]. In particular, conflicts between social workers and child welfare workers has been found to hinder interprofessional collaboration as well as that with parents [32]. In the former, some studies show that professionals regard working in pairs as a facilitator in the provision of integrated social care [41,44]. The involvement of two professionals in one family case (one for the parents and one for the children) can be a facilitator because the focus is often on the parents at the expense of their offspring [44].

#### Multidisciplinary teams

For both professionals and families, multidisciplinary expertise and the composition of teams [33,36,41] are potentially highly facilitative. militates against blind spots and enhances professional learning [41,43]. Multi-agency partnerships as a specific form of collaboration amongst professionals from different organizations reduce the probability of conflicts, strengthen relationships, and improve outcomes for families [40,41]. However, they require those involved to give up certain privileges that hinder the process and demand that managers address blind spots, which can be difficult [43]. Resistance to (integrated) collaboration and competition among organizations are barriers to integrated social care [30,31].

#### Coordination of care

A care coordinator is often assigned the formal task of maintaining an overview of the support process and encouraging and coordinating interprofessional collaboration [33]. Three studies note that professionals and family members appreciate care coordination as a facilitator in preventing or resolving conflicts between, for example, social and child welfare services [31,33,41].

In addition, care coordination implies a sense of mastery over the support process by professionals and families experiencing multiple and complex problems [31]. Care coordination is also found to improve interagency collaboration between different organizations, particularly if they share policies, protocols, and training opportunities [31].

#### Training and supervision

Several of the studies make a plea that professionals remain up to date through training and supervision because there is always the risk that their expertise will not be utilized when working within multidisciplinary teams [32,40,41]. What is more, through training and supervision, professionals can learn to become more resilient and maintain control over situations when they are supporting families.

#### Professional autonomy

Professional autonomy makes possible the provision of tailor-made guidance to families [31,40,41]. Too much autonomy, however, can make tasks more opaque [41]. While flexibility in the duration and intensity of care [31] is considered a facilitator for integrated social care [40], too much flexibility can lead to burnout [31,33]. High pressure and waiting lists have also been identified as barriers [33,41].

#### Roles and task structure

Professionals have identified the need for a broad assessment of the support needed by families, a continuous support pathway, and an ongoing evaluation of the support process within the families themselves as important facilitators [41]. Some studies show that clear agreements about tasks, roles, and responsibilities are also facilitative [33,41].

A potential barrier to integrated social care as a broad approach can arise when professionals provide support in areas outside their expertise and when they provide too much support for relatively trivial issues [41]. Professionals may not always have the skills necessary to provide the required support. As a result, they may experience a sense of loss of competence and control [41].

#### Accessibility of care

For professionals, being able to provide families with access to multiple providers through one organization facilitates integrated social care [43]. For the families themselves, this one point of entry may determine whether they have a positive experience of the process [33,36]. In addition, professionals see working at a co-location as an advantage because it creates a greater sense of familiarity, generates stronger relationships [33,41], and makes it possible to respond more quickly when support is needed [33,36,41].

Barriers to accessibility include a want of strict referral procedures [30,31,39,44]; by contrast, clear ones are facilitative [40,43]. Underdeveloped pathways for intra- and interagency collaborations are another barrier [31].

## Discussion

This scoping review aims to identify facilitators and barriers for families receiving integrated social care and for professionals providing such care.

### Key elements for families

Our review shows that multiple studies highlight that collaborate relation based on trust between families and professionals, crucial for providing integrated social care [31,32,34,35,37]. Also, others studies suggest that if there is sufficient trust, if information is not withheld and if the family is more involved in the assistance, an uncooperative and/or sceptical attitude of the family can be prevented [45]. Our review also indicates that family members often have a considerable level of distrust towards care services [29,30,31,32]. Reflecting on this issue, it becomes evident that efforts should be directed towards fostering and strengthening trust-based collaborative relationships between families and professionals.

In addition, shared decision making can enhance trust in supporting families [30,31,33]. An other study also reported that involving families in the decision-making process, professionals not only gain valuable insights into the family’s perspective but also empower families [46]. Another study show that shared decision making is an facilitator for family members to take an active role in defining their priorities [1]. As shared decision making enhances coping, problem-solving, and empowerment [45] and problem prioritization, it can be considered integral to integrated social care.

A systematic approach involving all family members should serve as a fundamental pillar in the provision of integrated social care to families [33,35,36,37,38,40,41]. We think that a systemic approach is necessary, but at the same time it is complicated by the current fragmented social care system. Preventing fragmentation by developing policy that prevents this fragmentation of care is therefore needed. This can be done, for example, by not only financing per field, discipline or specialization (monodisciplinary) but by allocating financial resources for multiple disciplines, fields and specializations (multidisciplinary) so that integrated policy can be made. In addition, multi-agency collaboration, where organizations work together to develop integrated social care, is also necessary. More research is needed on these aspects.

Explicitly involving the informal network can be supportive in avoiding fragmentation between those two sources of support [42]. However, one other study shows that the successful involvement of informal networks in formal social care has often limited attention in practice [45]. This underlines the need for a more concerted effort in practice to bridge the gap between formal and informal support network.

In addition, other studies shows that the informal network of these families cannot always contribute positively because these networks are often unstable or because there is a lack of positive parenting norms [47,48]. For this reason, we endorse that a tailor-made assessment must be done for insight in the added value of linking the formal care and the informal network. While, our study indicates the usefulness of informal networks, there is however also a lack of in-depth understanding of this phenomenon, so further research is needed.

### Key elements for professionals

For professionals, the wide variety and complexity of problems can make them feel overwhelmed [38]. In line with Lonne and colleagues [46] we argue that resilient professionals are better able to provide qualitative support in often difficult situations of these families [46]. Organizations must therefore ensure that the right conditions exist for strengthening the resilience of these professionals [46]. For delivering support, professionals need clarity about formal agreements on tasks, roles, and responsibilities to avoid overburdening [41]. Also, professional autonomy e.g., in making decisions, the professional needs space to do what is necessary in supporting these families [41]. Care providers face several barriers in their efforts to provide integrated social care. One of these is the fragmentation of services across different sectors and organizations [7,8,9,31]. A lack of collaboration between different service providers can result in gaps, duplications, and inconsistencies in support. Interprofessional collaboration can be a possible solution and strengthen resilience of professionals.

Interprofessional collaboration is an essential part of integrated social care. In this review, we identified several facilitators for interprofessional collaboration, including shared learning [32], multi professional decision-making [29,31], warm handoffs [33,41] and case discussions [41]. These facilitators play a crucial role in enhancing the capabilities of professionals, allowing for the collective deployment of expertise. This positive influence can significantly contribute to fostering successful interprofessional collaboration.

A specific form of interprofessional collaboration is working in pairs, the benefits of which include collaboration and mutual support in the form of feedback, debriefing, continuity of care, and the sharing of knowledge and expertise [41,44]. Working in pairs can be valuable by allowing one professional to concentrate on the children and their needs while the other focuses on the parents with their needs [44]. This division of attention ensures that the child or children receive adequate care and attention within the context of the family dynamic. Although research on this subject is limited, working in pairs could play a significant role in integrated social care for families. We urge future research into working in pairs.

While the goal of integrated care is to improve coordination and collaboration, it can also introduce additional challenges and stressors for professionals [32,41]. Therefore it is important to provide training and supervision opportunities for professionals, establishing robust information-sharing protocols that prioritize family privacy, and actively fostering partnerships with other organizations. Collaborative efforts are needed to cultivate a culture of collaboration and shared responsibility. Because families receive support from multiple professionals, future research should provide in-depth insight into effective elements and mechanisms for interprofessional collaboration with these families of interprofessional collaboration in supporting families is required.

### Strengths and limitations

The present study, which is the first scoping literature review of integrated social care, has several limitations. First, integrated social care is a broad and multifaceted concept that lacks a precise definition. Therefore, the studies we have identified may not always explicitly use the term integrated social care. To address this issue, we conducted a thorough search within the selected articles for components related to integrated social care based on the definition underlying the present study. In addition, the concept of families with multiple and complex problems is often operationalized or described differently [11].

Secondly, we report on barriers and facilitators that apply not only to integrated social care but also to the provision of integrated care in general, for instance, strengthening collaborative relationships, asking for feedback, the use of humour, and home visits. These facilitators and barriers are fundamental to the principles of integrated social care and are inherently part of it. Therefore, we did not differentiate these general facilitators from the more specific ones for integrated social care. Also, the studies were of a high quality, thereby ensuring the reliability of the evidence (Appendix 2).

## Conclusion

We found the key elements of integrated social care to be a systemic approach based on trust; shared decision-making; social networks; and coordinated care. Shared decision-making helps to establish a systemic approach and empowers all family members (including the children). This allows them maximum control over the support process and respect for their autonomy, If a family does not have a supportive informal network, this can give its members more agency. Finally, care coordination can help to prevent fragmentation, especially when its implementation involves a care plan. Families often face multiple and complex problems and interact with various professionals, it is crucial that there be integrated collaboration between the families and these professionals within social care.

## Additional Files

The additional files for this article can be found as follows:

10.5334/ijic.7768.s1 Appendix 1. Search overview databases.

10.5334/ijic.7768.s2 Appendix 2. Methodological appraisal of the studies: qualitative.
