# Supplementary material for: Facilitators and Barriers in Integrated Social Care for Families Facing Multiple and Complex Problems: A Scoping Review
Source: Int J Integr Care. 2024 Aug 7;24(3):13. doi: 10.5334/ijic.7768 (PMC11312847; doi:10.5334/ijic.7768)
Supplement: Appendix 1. — Search overview databases. [file ijic-24-3-7768-s1.pdf]

Appendix 1. search overview databases		**Web of Science**	**PsycINFO**	**Psychology and Behavioural Sciences Collection**	**CINAHL**	**MEDLINE**	**PubMed**
**Key words**	Search string	Date:<br>28-09-2023	Date:<br>28-09-2023	Date:<br>28-9-2023	Date:<br>28-9-2023	Date:<br>28-9-2023	Date:<br>28-9-2023
**(Population)**<br>**Families with multiple and complex problems**	(“multi-problem famil*” OR “multiproblem famil*” OR “famil* with multi-problem*” OR “famil* in multiple problem*” OR “multi-stressed famil*” OR “multi-crisis famil*” OR “multi-assisted famil*” OR “multiproblem famil*” OR “vulnerable famil*” OR “families with longstanding and complex*” OR “famil* with complex problems”)	140	115	28	124	99	92
**(Intervention)**<br>**Integrated social care**	AND (fragmentation* OR fragmented* OR integrated* OR integral* OR “multi-agency” OR “multi agency” OR interprofessional* OR collaborat* OR generalis* OR care coordinat* OR coordinat* OR multiprofessional* OR multi-disciplinary*)						
**(Comparison)**	-						
**(Outcome)**	-						
	Searchstring for PubMed						
**(Patient)**<br>**Families with multiple and complex problems**	“multi-problem famil*”[tiab] OR “multiproblem famil*”[tiab] OR “families with multi-problem*”[tiab] OR “families in multiple problem*”[tiab] OR “multi-stressed famil*”[tiab] OR “multi-crisis famil*”[tiab] OR “multi-assisted famil*”[tiab] OR “vulnerable famil*”[tiab] OR “families with longstanding and complex”[tiab] OR “families with complex problems”[tiab]						
**(Intervention)**<br>**Integrated social care**	AND fragmentation*[tiab] OR fragmented*[tiab] OR integrated*[tiab] OR integral*[tiab] OR “multi-agency”[tiab] OR “multi agency”[tiab] OR interprofessional*[tiab] OR collaborat*[tiab] OR generalis*[tiab] OR care coordinat*[tiab] OR coordinat*[tiab] OR “multi professional*” [tiab] OR multi-disciplinary*[tiab]						
**(Comparison)**	-						
**(Outcome)**	-						
							Tot. 598
