# Supplementary material for: Facilitators and Barriers in Integrated Social Care for Families Facing Multiple and Complex Problems: A Scoping Review
Source: Int J Integr Care. 2024 Aug 7;24(3):13. doi: 10.5334/ijic.7768 (PMC11312847; doi:10.5334/ijic.7768)
Supplement: Appendix 2. — Methodological appraisal of the studies: qualitative. [file ijic-24-3-7768-s2.pdf]

Appendix 2. Methodological appraisal of the studies: qualitative

	Key issues in critical appraisal of qualitative research						
	Clear purpose	Appropriate rationale	Clear outline of the conceptual framework	Clarity re. data analysis and purpose of study	Clarity re. reliability and validity of data collection, analysis, and interpretation	Clear progression from research question to conclusions	Overall judgement of methodological quality
1. Sousa, L. (2005). Building on personal networks when intervening with multiproblem poor families.	+	+	±	+	+	+	Good
2. Thoburn et al. (2013). The place of “think family” approaches in child and family social work: Messages from a process evaluation of an English pathfinder service.	+	+	+	+	+	+	Good
3. Onyskiw et al. (1999). Formative evaluation of a collaborative community-based child abuse prevention project.	+	+	+	+	+	+	Good
4. Nooteboom et al. (2020a). An integrated approach to meet the needs of high-vulnerable families: a qualitative study on integrated care from a professional perspective.	+	+	+	+	+	+	Good
5. Lawick et al. (2008). Building bridges: home visits to multi stressed families where professionals help reached a deadlock.	-	±	±	±	±	±	Mediocre
6. Nadeau et al. (2012). Partnership at the forefront of change: documenting the transformation of child and youth mental health services in Quebec.	+	+	+	+	±	±	Good
7. Sousa L, & Rodrigues S. (2009). Linking formal and informal support in multiproblem low-income families: the role of the family manager.	+	+	+	±	±	+	Good
8. Nooteboom et al. (2020b). What do parents expect in the 21st sentury? A qualitative analysis of integrated youth care.	+	+	+	+	+	+	Good
9. Tennant et al. (2020). A critical realist evaluation of an integrated care project for vulnerable families in Sydney, Australia.	+	+	+	+	+	+	Good
10. Eastwood et al. (2020a). Refining program theory for a place based integrated care initiative in Sydney, Australia.	+	+	+	+	+	+	Good

11. Eastwood et al. (2020b) Care coordination for vulnerable families in the Sydney local health district: what works for whom, under what circumstances, and why?	+	+	+	±	+	+	Good
12. K. Morris (2010). Troubled families: vulnerable families' experiences of multiple service use.	+	+	+	+	+	+	Good

Methodological appraisal of the studies: quantitative

	Methodological quality (items)																
Study	Randomization	Blinded incl.	Pat/clin blinding	Resear-cher blinding	Comparable groups		Sufficient follow-up		Analy-zed in random group	Equal treatm. Contr/ interv.	Selective Publica-tion	Excl. influence Interest parties	Agree-ment own patients	Feasibi-lity own practice	(Dis)Advantages own patients	Expecta-tions preferences own patients	**# pos items of total**
					Yes	Analysis if no	Yes	Analysis if no									
Bachler et al. (2015; RCT)				X			X				X	X	X	X		X	8/14
Bachler et al. (2017; validation study)							X		X	X	X	X	X	X			7/14
Tausendfreund et al. (2014; prospective study)							X		X		X	X	X	X			6/14
Serbati et al. (2016; pre- and post-test)			X		X		X		X	X	X	X	X		X	X	10/14
